# Hindlimb Immobilization Increases IL-1β and Cdkn2a Expression in Skeletal Muscle Fibro-Adipogenic Progenitor Cells: A Link Between Senescence and Muscle Disuse Atrophy

**DOI:** 10.3389/fcell.2021.790437

**Published:** 2022-01-03

**Authors:** Emily Parker, Andrew Khayrullin, Andrew Kent, Bharati Mendhe, Khairat Bahgat Youssef El Baradie, Kanglun Yu, Jeanene Pihkala, Yutao Liu, Meghan McGee-Lawrence, Maribeth Johnson, Jie Chen, Mark Hamrick

**Affiliations:** ^1^ Department of Cellular Biology and Anatomy, Medical College of Georgia at Augusta University, Augusta, GA, United States; ^2^ Faculty of Science, Tanta University, Tanta, Egypt; ^3^ Flow Cytometry Core Facility Research Laboratory Director, Medical College of Georgia at Augusta University, Augusta, GA, United States; ^4^ Division of Biostatistics and Data Science, DPHS, Medical College of Georgia at Augusta University, Augusta, GA, United States

**Keywords:** atrophy, SASP, RNA-seq, disuse, progenitor cell

## Abstract

Loss of muscle mass and strength contributes to decreased independence and an increased risk for morbidity and mortality. A better understanding of the cellular and molecular mechanisms underlying muscle atrophy therefore has significant clinical and therapeutic implications. Fibro-adipogenic progenitors (FAPs) are a skeletal muscle resident stem cell population that have recently been shown to play vital roles in muscle regeneration and muscle hypertrophy; however, the role that these cells play in muscle disuse atrophy is not well understood. We investigated the role of FAPs in disuse atrophy *in vivo* utilizing a 2-week single hindlimb immobilization model. RNA-seq was performed on FAPs isolated from the immobilized and non-immobilized limb. The RNAseq data show that IL-1β is significantly upregulated in FAPs following 2 weeks of immobilization, which we confirmed using droplet-digital PCR (ddPCR). We further validated the RNA-seq and ddPCR data from muscle *in situ* using RNAscope technology. IL-1β is recognized as a key component of the senescence-associated secretory phenotype, or SASP. We then tested the hypothesis that FAPs from the immobilized limb would show elevated senescence measured by cyclin-dependent kinase inhibitor 2A (Cdkn2a) expression as a senescence marker. The ddPCR and RNAscope data both revealed increased Cdkn2a expression in FAPs with immobilization. These data suggest that the gene expression profile of FAPs is significantly altered with disuse, and that disuse itself may drive senescence in FAPs further contributing to muscle atrophy.

## Introduction

The muscle atrophy that occurs with aging, bed rest and spinal cord injury is associated with decreased independence and poor quality of life. Maintenance of skeletal muscle mass and function relies on two resident muscle stem cell populations: satellite cells (SCs) and fibro-adipogenic progenitor cells (FAPs). SCs are muscle progenitor cells that can differentiate into myoblasts and ultimately myotubes. Recent studies show that SCs and FAPs interact closely with one another during the process of muscle regeneration and, in the absence of FAPs, muscle regeneration is impaired (Wosczyna et al., 2019). FAPs are a group of mesenchymal precursor cells that are normally quiescent but become active with muscle injury (Biferali et al., 2019). These cells express PDGFRα+, Sca1+, and CD34^+^, but not CD31, CD45, CD11b, or α7-integrin (Joe et al., 2010; Uezumi et al., 2010; Wosczyna et al., 2012; Lukjanenko et al., 2019). The number of FAPs in normal muscle tissue can be two-to three-times that of satellite cells (∼5–15% of the muscle cell population; Contreras et al., 2019a; Contreras et al., 2019b; De Micheli et al., 2020a; De Micheli et al., 2020b; Lee et al., 2020; Santini et al., 2020) whereas satellite cells normally represent only ∼2–5% of muscle cells (Shi and Garry, 2006). FAPs impact neighboring satellite cells and myocytes via the secretion of factors such as IL-4, IL-15, IL-6 that are collectively referred to as the FAP secretome (Joe et al., 2010; Heredia et al., 2013; Kang et al., 2018; Stumm et al., 2018; Biferali et al., 2019).

FAPs can differentiate to form adipocytes or fibroblasts under various conditions. Fibrogenic differentiation of FAPs is stimulated by Wnt-TGFβ signaling in dystrophic muscle and in amyotrophic lateral sclerosis (Biressi et al., 2014; Gonzalez et al., 2017; Mazala et al., 2020). Fatty infiltration of muscle, or myosteatosis, with aging is accompanied by changes in the gene expression of FAPs. Specifically, aged FAPs increase expression of lipid metabolism pathways such as PI3K-AKT and MAPK signaling pathways (Xu et al., 2021). Aged FAPs express genes that increase collagen deposition and downregulate genes that stimulate cell differentiation (Kimmel et al., 2021). Together these studies suggest that FAPs play a key role in supporting normal muscle repair and regeneration as well as contributing to muscle dysfunction with aging and disease (Parker and Hamrick, 2021).

Muscle atrophy occurs in a variety of settings such as spinal cord injury, aging in the form of sarcopenia, bedrest following hip fracture, and prolonged exposure to microgravity with spaceflight. Sarcopenia in particular affects one out of three individuals over the age of 60 and more than half of individuals over age 80 (von Haehling et al., 2010). A critical gap in the treatment and prevention of muscle loss with disuse is a poor understanding of the cellular and molecular mechanisms by which muscle progenitor cells contribute to muscle atrophy. Here we investigate this question by examining gene expression changes in FAPs with hindlimb immobilization.

## Materials and Methods

### Immobilization Model

**FIGURE 1:**
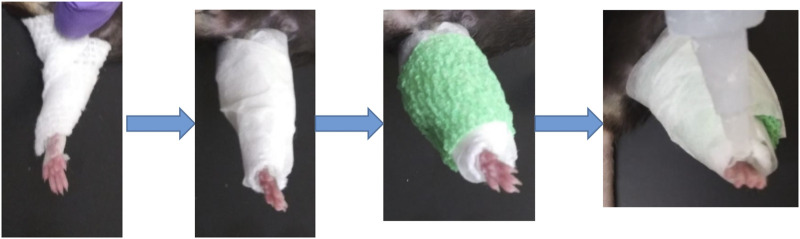
Schematic of single hindlimb immobilization model. Immobilization of the left hindlimb of the mouse. Gauze was secured to the pad of the mouse foot used tape and wrapped securely around the leg. Tape was then wrapped around the gauze, followed by vet wrap. **(A)** layer of tape and a drop of superglue at the top of the foot finished the cast.

Adult (12 m) male and female C57/BL6 mice (9 males, five females) were used in this study for single hindlimb immobilization. Mice had ad libitum access to food and water and were on a normal light cycle (6am-6pm light/6pm-6am dark). All procedures were reviewed and approved by the Augusta University IACUC. The left hindlimb of each mouse was immobilized for 2 weeks using an adapted version of a Velcro cast (Aihara et al., 2017). Cast padding (McKesson, 16–010), connected to the pad of the foot by surgical tape, was wrapped from the pad of the foot around the hindlimb several times (Figure 1). This padding was secured in place with a layer of surgical tape. A drop of super glue was added at the top of the foot to complete the cast. Replacement of the cast was performed as needed throughout the 2 weeks, usually every 2–3 days. After 2 weeks of immobilization the animals were euthanized following IACUC approved procedures.

### Quadriceps Histology

Immediately after euthanization, quadriceps muscles were dissected free, weighed, and placed in 10% buffered formalin. Muscles were removed from fixation after 24 h and stored in 70% ethanol. Samples were paraffin embedded, cut in cross section, and trichrome stained to facilitate measurement of fiber size. Fiber size was calculated by taking images from standardized areas of each cross section at ×20 magnification using a brightfield Leica DMCS microscope outfitted with a micropublisher six camera (QImaging). Ocular software (QImaging) was then used to take images of each sample which were then analyzed with ImageJ to calculate the area of individual muscle fibers. Thirty muscle fibers were measured per sample to calculate an average fiber size per muscle. Slides were stained for H&E using standard protocol and images were obtained using both 20x and 40x magnification.

### Fluorescence Activated Cell Sorting (FACs) for FAP Cell Isolation

A total of six 12-month-old C57/BL6 mice (three males and three females) mice were used for FACs sorting. The mice were immobilized for 2 weeks as described above. Promptly after euthanization quadriceps mass was measured, leg muscles were isolated, and muscles were then dissociated with forceps and placed into an enzymatic mixture (dispase II 2.5 U/mL, Roche, collagenase B 0.2% Roche, and MgCl2 5 mM) resting on ice. The mixture was placed in a water bath (37°C) for 45 min, briefly vortexing every 15 min to ensure even digestion. The samples were filtered using a 100 and 40 µm nylon cell strainer (BD Biosciences). The cells were then stained with the following antibodies: CD45 (Invitrogen, MCD4528), CD31 (Invitrogen, RM5228), CD11b (Invitrogen, RM2828), CD34 (BD Biosciences, 551,387), Ly-6A-Ly6E (BD Biosciences, 561,021), *α*7-integrin (R&D, FAB3518N), and for PDGFRa either CD140a (eBioscience, 17-1,401-81) or CD140a MicroBead Kit (Miltenyl Biotec, #130-101-547) was used. The cells were then sorted using BD Biosciences FACSCAria II SORP based on: CD31-/CD11b-/CD45-/Sca1+/CD34+/(CD140a) PDGFR-*α*+. The cells were sorted into 500 µL media, pelleted, and all but 50 µL of media was removed. 500µL of TRIzol was added to the samples, frozen on dry ice and then stored at −80. For those sorted with a bead kit, the cells were sorted according to: CD31-/CD11b-/CD45-/Sca1+/CD34+ and sorted into 500 µL media following manufacturer specifications and samples stored in TRIzol as described above (Supplementary Figure S1).

### Total RNA-Seq and Subsequent Analysis

Total RNA was extracted from sorted cells using Single Cell RNA Purification Kit (NORGEN) according to the recommended procedure. RNA quality was assessed using Agilent 2,100 Bioanalyzer with RNA 6000 Pico kit (Agilent Technologies) and assured of RNA Integrity Number (RIN) greater than 5.0. Total RNA from 12 samples were processed for cDNA library preparations using SMARTer Stranded RNA-seq kit v2 Pico Input (Clontech_TaKaRa). Briefly, 1 ng of total RNA was fragmented based on the quality of the RNA input and converted to cDNA fragments. These cDNA fragments then had the addition of a single “A” base and subsequent ligation of the adapter. Following purification, the cDNA libraries were treated with ZapR and R-Probes v2 (Clontech_TaKaRa) to deplete rRNA (18S and 28S). The rRNA depleted libraries were enriched with PCR and purified to create the final libraries. Each library was examined by Bioanalyzer and Qubit HS DNA kit (Thermofisher) to evaluate library quality and quantity, respectively. A total of 12 libraries were pooled and run on an Illumina NextSeq500 sequencer with high output using 150-cycle for 75bp paired-end at the Integrated Genomics Shared Resource in the Georgia Cancer Center of Augusta University. BCL files generated from the NextSeq500 are converted to FASTQ files for downstream analysis. The pair-end raw sequence reads from 12 RNA samples were transferred to Partek Flow server via SFTP producing 12 paired RNA-seq samples. The analysis was performed using Partek Flow RNA-Seq tool. After pre-alignment QA/QC check and base trimming the sequencing reads were aligned to mm10 genome using the Bowtie2 aligner. These aligned reads were then quantified to annotation model mm10-emsembl-release-97 using Partek E/M algorithm, followed by data normalization (offset, scaling, and log base two transformation) to generate sequence counts of 23,416 unique mouse genes for down-stream differential expression analysis. Pre-alignment, the total reads ranges from 33 + million to 44 + million reads per sample with QA/QC score of at least 33.83 per sample. Through pre-alignment QA/QC check and base trimming the sequencing reads were aligned to mm10 genome using the Bowtie2 aligner. The alignment rate ranges from 31 to 61% per sample with post-alignment QA/QC score of at least 34.14.

### Droplet Digital PCR of Target Genes

Droplet digital PCR (ddPCR) was performed using approximately 10 ng total RNA from isolated FAPs transcribed into cDNA using the iScript cDNA Synthesis Kit (Biorad) according to the manufacturer’s instructions. The ddPCR assay utilized a QX200 ddPCR Supermix for Probes (no dUTP) from Bio-Rad. The PCR products were quantified using the Bio-Rad QX200 droplet reader and subsequently analyzed with the QuantaSoft software as previously described (Helwa et al., 2017). On average 15,000 droplets were obtained during the process for each sample, with a requirement of 10,000 droplets needed for a sample to be analyzed. There also had to be a clear separation of background (noise) droplets and the signal droplets analyzed. Probe assays for IL-1β (dMmuEG5065947, Biorad), Actb (dMmuEG5193531, Biorad) and Cdkn2a (dMmuEG5193677) were used to detect gene expression.

### qRT-PCR

Gastrocnemius muscles from twelve immobilized and twelve nonimmobilized samples were sonicated, and RNA was isolated using the TRIzol reagent (Invitrogen) according to the manufacturer’s instructions. Total RNA was isolated using a RNeasy Mini Kit (Qiagen). 1µg of total RNA was then reverse transcribed using the iScript cDNA Synthesis Kit (BioRad). PCR was then run using primers for UBC (Forward: 5′-AGC​CCA​GTG​TTA​CCA​CCA​AG-3′, R: 5′-ACC​CAA​GAA​CAA​GCA​CAA​GG-3′), ActB (F: 5′-AGC​CAT​GTA​CGT​AGC​CAT​CC-3′, R: 5′-GCT​GTG​GTG​GTG​AAG​CTG​TA-3′) and IL1β (F: 5′-GCA​ACT​GTT​CCT​GAA​CTC​AAC​T-3′, R: 5′-ATC​TTT​TGG​GGT​CCG​TCA​ACT-3′).

### RNAscope Staining

Slides were submerged in xylene and 100% EtOH then treated with hydrogen peroxide for 10 min at room temperature. Slides were placed in pretreatment (ACD Bio) and boiled at 99°C for 30 min using a rice cooker. The slides were then incubated with protease plus (ACD Bio) for 30 min at 40°C in the EZ Hybrid Oven (ACD Bio). RNAscope multiplex assay protocol was then performed according to manufacturer instructions. In brief, the probe mixtures (ACD Bio) were placed on the slides for a 2-h incubation at 40°C. Probes used in this study were for IL-1β (NM_008,361.3, 2–950), Cdkn2a (NM_009877.2, 35-886) and PDGFRα (NM_011058.2, 223-1161). Positive and negative control probes were provided by ACD Bio. Afterwards, the slides were incubated overnight at 4°C in 5xSSC buffer (Millipore Sigma). The slides were then incubated with AMP1, AMP2 and AMP3 (ACD Bio) at 40C. This was followed by incubation of HRP-C1 (ACD Bio), followed by the corresponding opal dye, followed by HRP block (ACD Bio). This was repeated for C2 and C3. Opal dyes 570, 620 and 690 (Akoya Biosciences) were used and diluted in TSA dilution buffer (ACD Biosciences). Once complete, mounting media with Dapi (Vector Labs) was added and the slide covered with a cover slip. Slides were then imaged using a Leica Stellaris confocal microscope at the Cell and Tissue Imaging Core Laboratory of Augusta University.

### Data Analysis

For differential analysis of RNA-seq data normalized gene counts were analyzed through a 2 × 2 ANOVA model (with leg and gender as the two factors) in Partek Flow. A *p*-value of 0.05 was used as the cut-off value for the factor leg with absolute fold change between left and right legs being at least 2-fold without interaction effect between leg and gender factors. Functional enrichment analysis of the RNA-seq data was performed using ToppFun analysis (Chen et al., 2009) of the top 25 differentially (increased) expressed genes. ddPCR results were analyzed using Exact Wilcoxon rank sum two sample test statistic with normalization of the gene of interest using the mean expression of ActB for each group. The normalized expression values were used in the statistical analysis. Paired t-tests were used to compare quadriceps weight, relative percentage weights and FACs sorted FAP cell numbers. Unpaired t-tests were used for the PCR analysis of IL-1β in whole muscle lysate. GraphPad Prism (GraphPad Software, San Diego, California, United States, version 8.0.0) was used for analysis unless otherwise indicated.

## Results

### Two-Week Single Hindlimb Immobilization Induces Significant Muscle Atrophy

**FIGURE 2:**
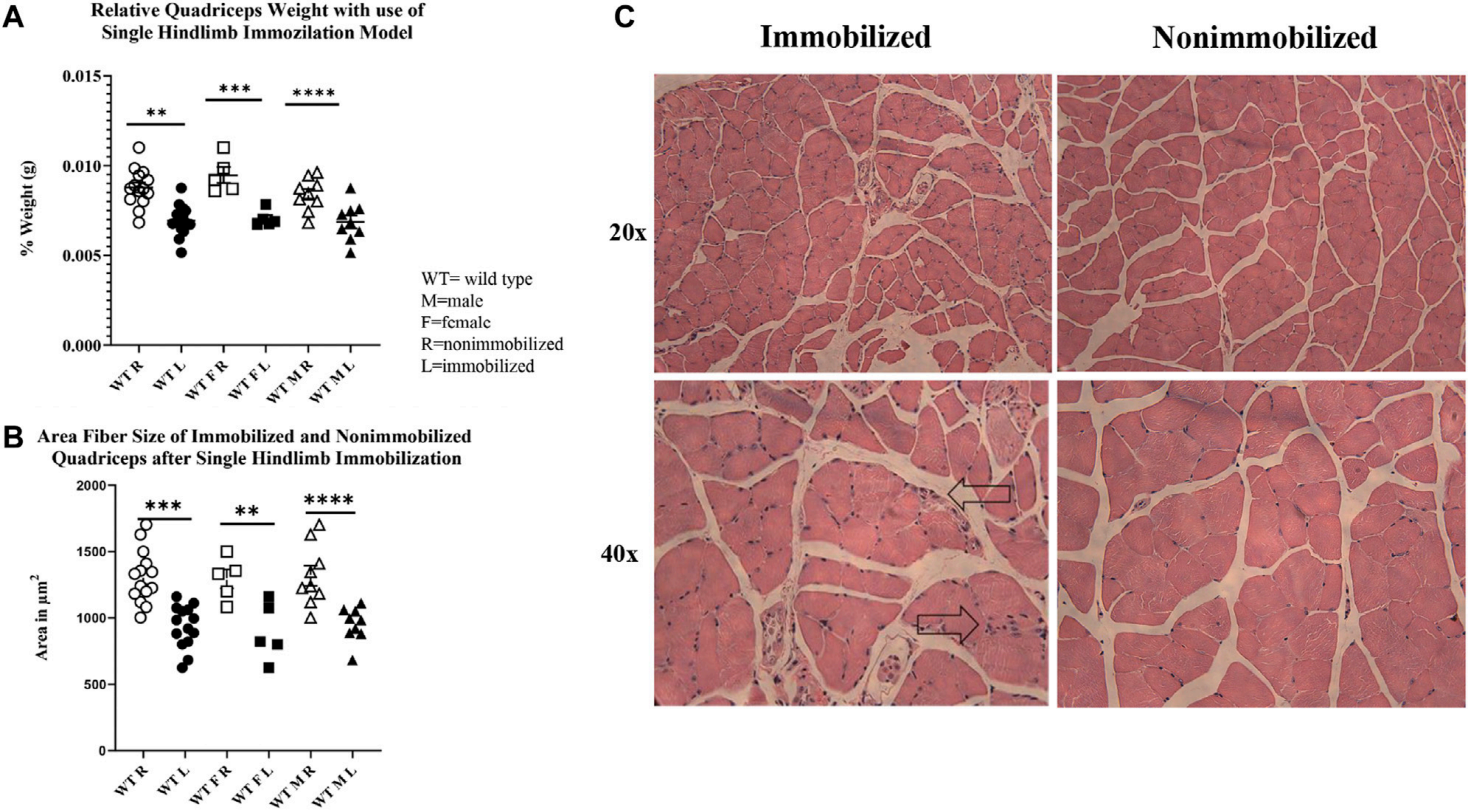
Muscle atrophy induced by 2-week single hindlimb immobilization. **(A)** Relative weights of quadriceps normalized to the mouse body weights. Significant reduction in relative quadriceps weight is seen in male, female, and combined groups. **(B)** Change in fiber size area of immobilized quadriceps (L) compared to nonimmobilized (R). Significant loss of fiber size area is seen in both the males, females, and combined groups. **(C)** H&E images of immobilized and nonimmobilized quadriceps muscle are shown at both 20x and 40x objectives. Black arrows highlight potential immune cell infiltration as indicated by the multiple nuclei (n.s *p* > 0.05, *p ≤ 0.05, **p ≤ 0.01, ***p ≤ 0.001, ****p ≤ 0.0001).

The 2-week single hindlimb immobilization period resulted in a significant loss of quadriceps muscle mass (*p* < 0.0001) and quadriceps fiber size (*p* < 0.0001) in both sexes (Figures 2A,B). There was not a significant difference detected in overall body weight among all mice (pooled data), although a loss of weight was seen in male mice (*p* < 0.05). These results suggest that the single hindlimb immobilization model used in this study can induce significant muscle atrophy over a two-week timeframe. This muscle atrophy can be visualized with smaller fibers in the H&E images in the immobilized muscle compared to the nonimmobilized muscle (Figure 2C).

### FAP Cell Number is Not Altered With Immobilization When Normalized to Total Cell Count

Total counts of FAP cell number were calculated during FACS sorting. Analysis of the counts showed that both FAP cell number and total cell number tended to be lower in the immobilized limb (Supplementary Table S2), so that FAP cell number normalized to total number of cells did not differ significantly between the immobilized and nonimmobilized limbs (*p* = 0.12).

### Muscle Atrophy Increases Expression of IL-1β in FAPs

**FIGURE 3:**
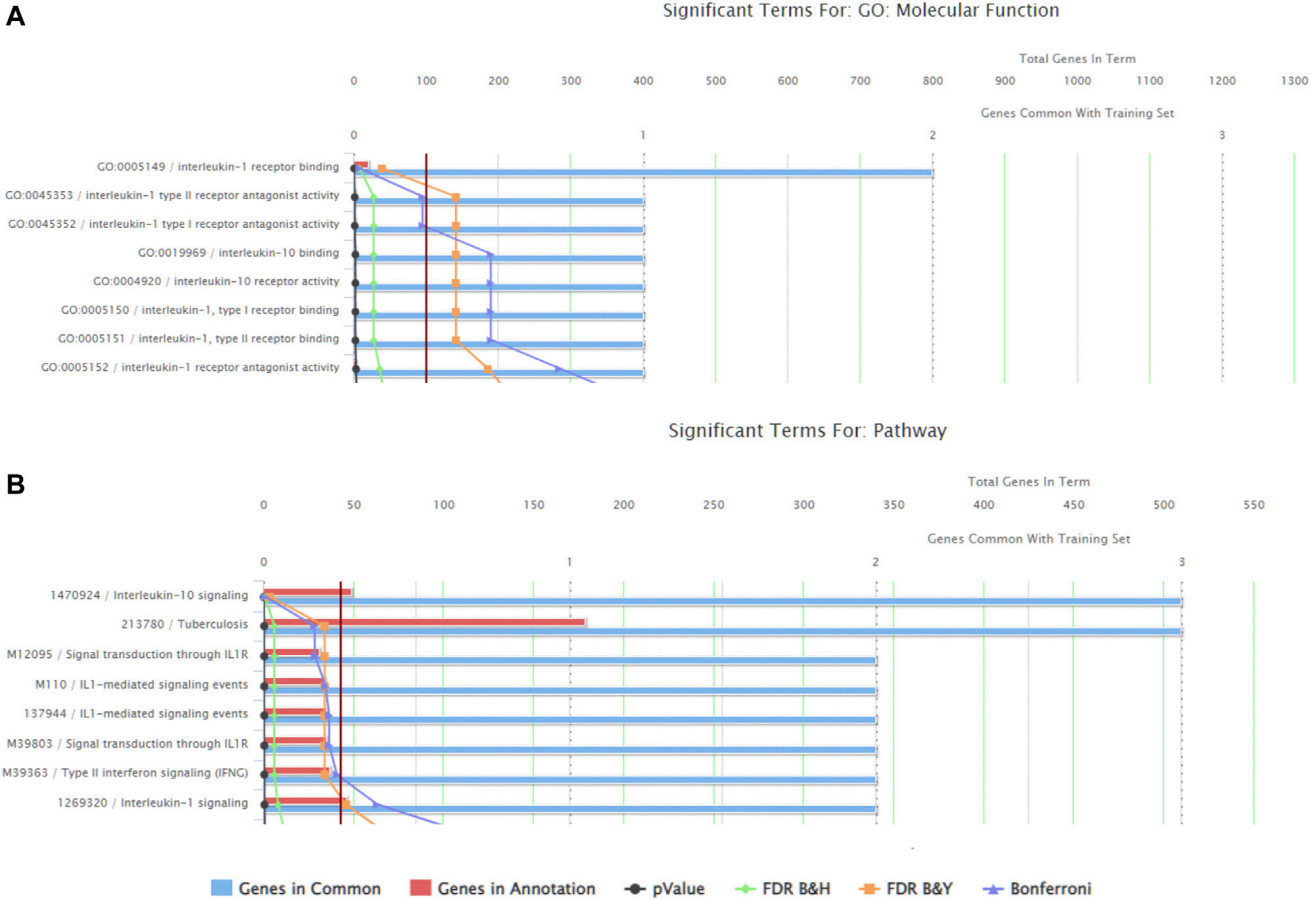
ToppFun functional enrichment analysis of the 25 most upregulated genes. **(A)** Top five molecular functions that are affected by the genes input in the system. Three of the five categories are IL-1β related, indicated that this gene is playing a role in the molecular functions being altered with atrophy. **(B)** Pathways that are significantly affect with the disuse model by the genes input in the system. Three of the five pathways are again related to IL-1 receptor signaling. Together this data suggests the importance of IL-1β.

There were a total of 919 genes differentially expressed with IL-1β identified as the gene whose fold-change (positive) was the greatest between muscle from the left (immobilized) and right limbs (Table 1). Functional enrichment analysis of the RNA-seq data also revealed changes in IL-1 signaling for both molecular functions (Figure 3A) and pathways (Figure 3B) with hindlimb immobilization.

**TABLE 1:** Top ten most upregulated genes. The top ten most upregulated genes are listed here with their corresponding *p*-values and fold changes from RNAseq results.

Gene symbol	*p*-value (Left vs. Right)	Fold change (Left vs. Right)
Il1b	2.00E-03	3.36E+04
Mmp13	3.95E-04	1.94E+04
Il1rn	8.88E-04	1.20E+04
Npy2r	2.19E-07	1.14E+04
Ciita	2.87E-03	1.05E+04
Il10ra	7.91E-10	9.86E+03
Pcdha11	2.21E-08	8.00E+03
Gm9025	1.68E-03	7.98E+03
Mtmr7	7.84E-04	7.91E+03

**FIGURE 4:**
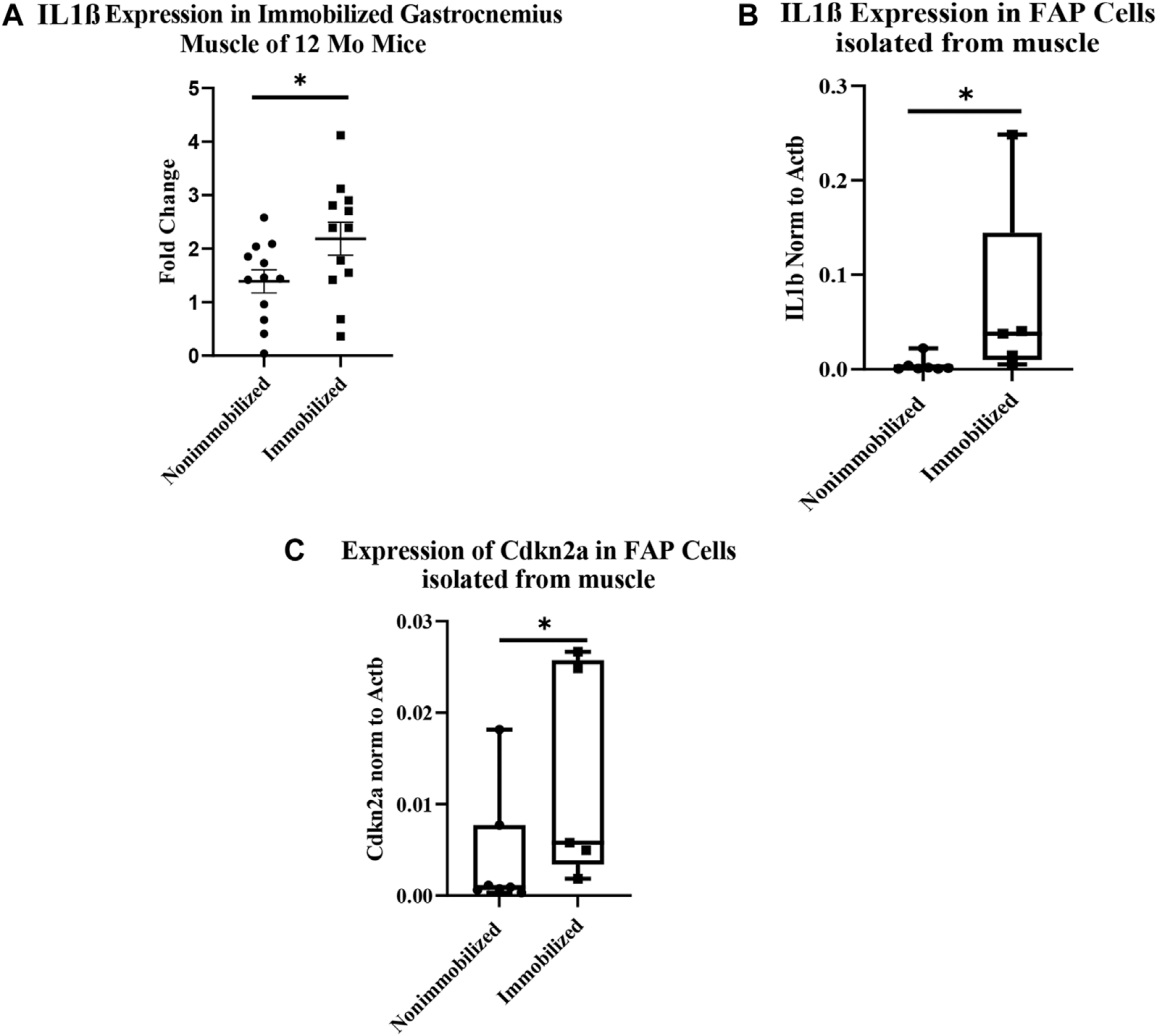
Muscle atrophy increases IL-1β expression and induces senescence in FAP cells. **(A)** IL-1β expression increased in immobilized muscle compared to nonimmobilized muscle (*p* < 0.05) **(B)** ddPCR results on the samples used for RNAseq validates the upregulation of IL-1β (*p* < 0.05). **(C)** ddPCR results show a significant increase in Cdkn2a expression in FAP cells isolated from immobilized muscle compared to nonimmobilized muscle (*p* < 0.05).

The qRT-PCR analysis of cDNA from whole immobilized and nonimmobilized muscle showed a significant increase in IL1β compared to the nonimmobilized muscle (*p* < 0.05, Figure 4A). The ddPCR analysis of the samples used for RNAseq confirmed the increase seen in IL1β expression among FAPs (*p* = 0.0051, Figure 4B). ddPCR was also performed for Cdkn2a expression as a marker of senescence in FAPs isolated from immobilized and non-immobilized quadriceps. Results showed a significant increase in Cdkn2a in FAPs isolated from the immobilized limb (Figure 4C). A full list of the 919 differentially expressed genes can be found in Supplementary Table S1.

### Immobilized Muscle Shows Co-localization of IL-1β, Cdkn2a and PDGFR*α*

**FIGURE 5:**
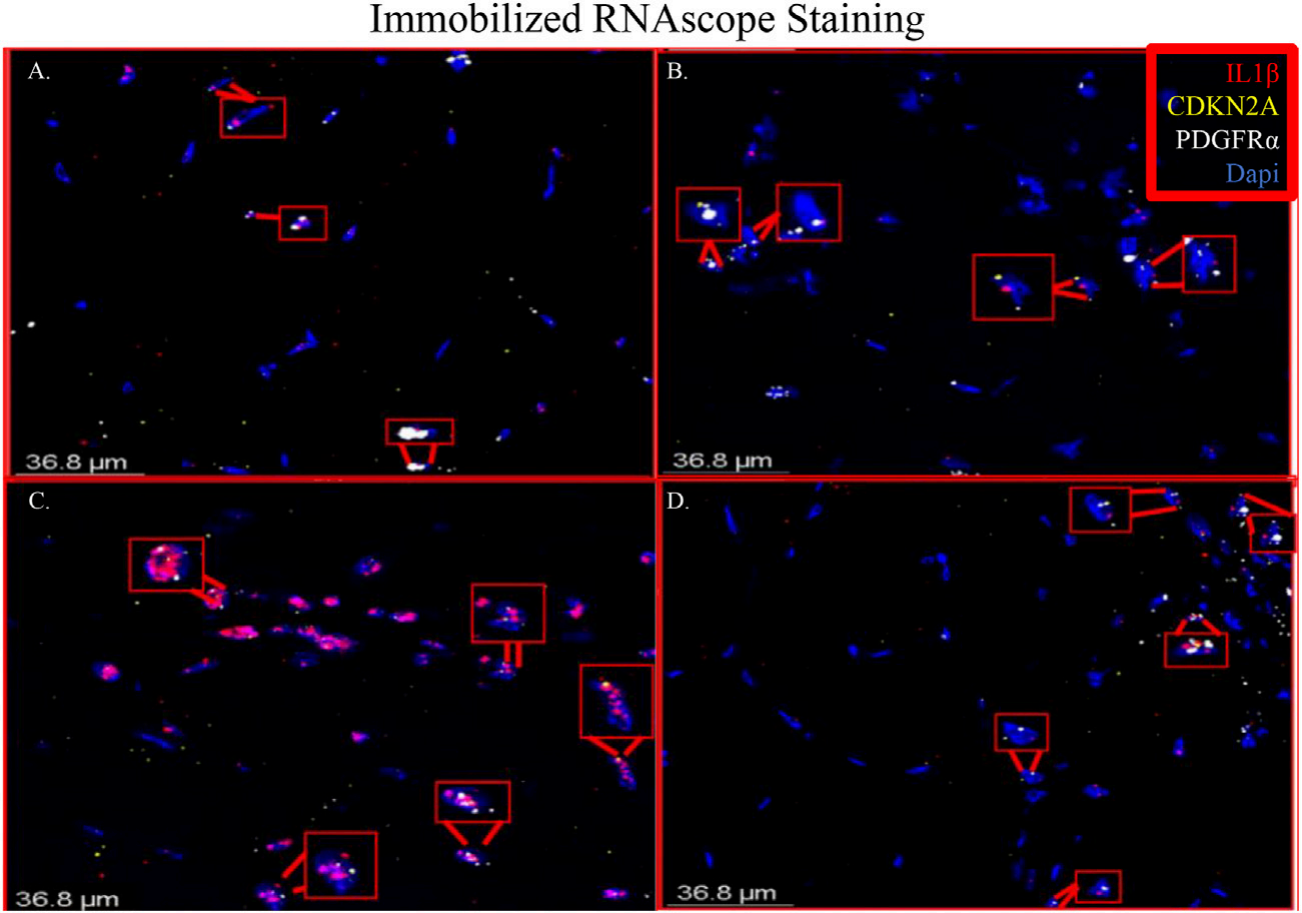
Merged RNAscope images of immobilized muscle. The merged RNAscope images of immobilized muscle with probes for IL-1β (red), Cdkn2a (yellow) and PDGFRα (white) staining are shown. Staining shows co-localization of PDGFRα with both IL-1β and Cdkn2a as indicated with the red boxes. Panel A corresponds to row one of Supplementary Figure S2, Panel B to row 2, Panel C to row 3 and Panel D to row 4.

**FIGURE 6:**
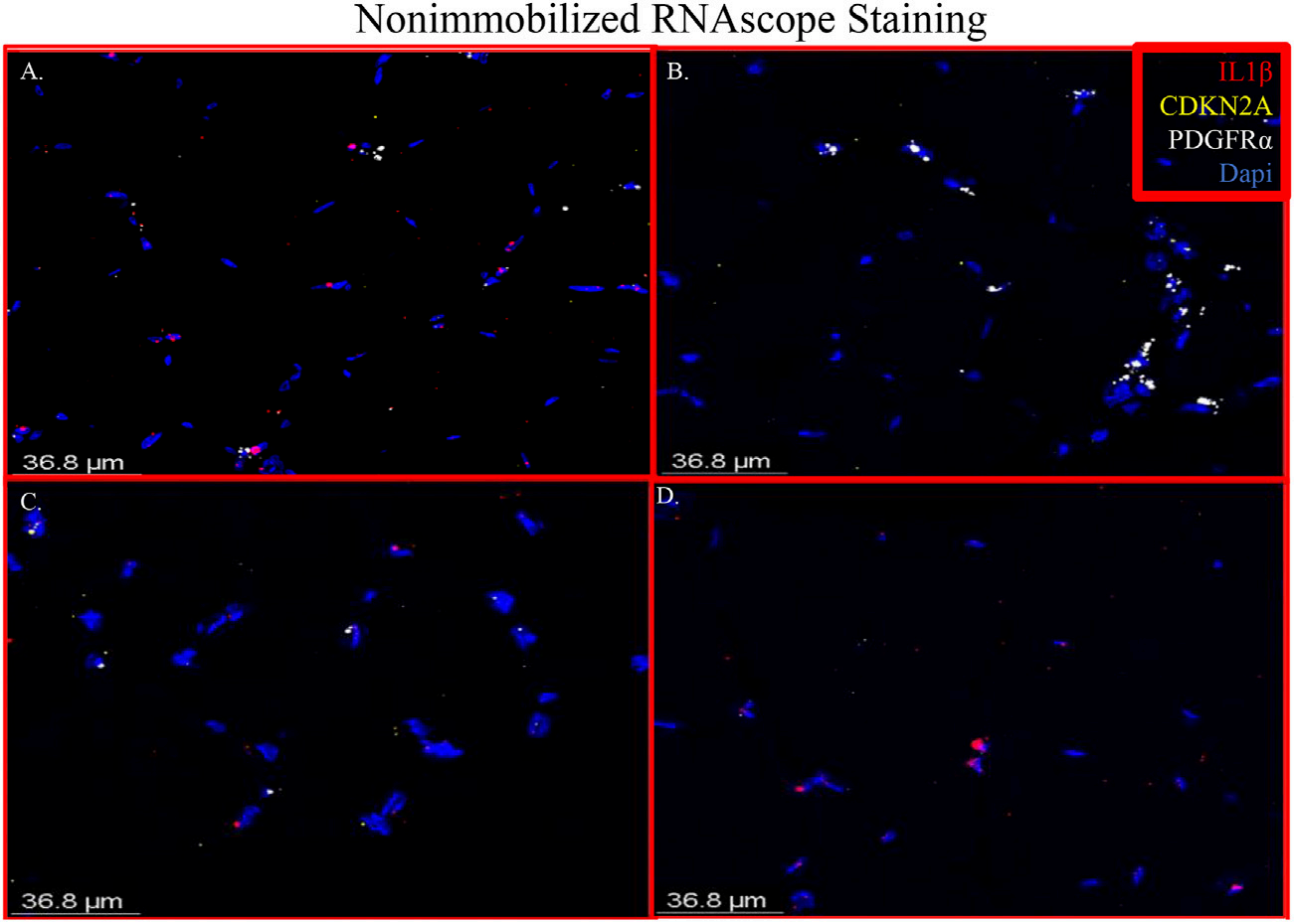
Merged RNAscope images of nonimmobilized muscle. The merged RNAscope images of the nonimmobilized muscle counterparts to Figure 5. The same probes for IL-1β (red), Cdkn2a (yellow) and PDGFR*α* (white) are used. Panel A corresponds to row one of Supplementary Figure S2, Panel B to row 2, Panel C to row 3 and Panel D to row 4.

RNAscope images show co-localization of IL-1β and PDGFRα in sections of immobilized quadriceps muscle (Figure 5 and Supplementary Figure S2). Co-localization of PDGFRα and Cdkn2a was also seen in immobilized muscle (Figure 5 and Supplementary Figure S2). In the non-immobilized contralateral control limbs, there was little to no co-localization seen, confirming that the immobilization treatment is inducing the expression of IL-1β and Cdkn2a in PDGFRα+ cells (Figure 6 and Supplementary Figure S3). These results support the ddPCR, PCR and RNAseq data showing that PDGFRα+ FAP cells increase the expression of these two genes after 2 weeks of immobilization. Negative control staining can be found in Supplementary Figure S4 (Supplementary Figure S4).

## Discussion

Our RNAseq analysis showed that a total of 919 genes were differentially expressed in FAPs after immobilization, and the most differentially expressed of these genes was the pro-inflammatory cytokine IL-1β. Enhanced IL-1β signaling in FAPs with unloading is further supported by our finding that the second most-highly expressed gene in FAPs after unloading is MMP-13, which is well-known to be stimulated by IL-1β (e.g., Vincenti and Brinckerhoff, 2001) in the settings of both aging and senescence (Forsyth et al., 2005). IL-1β activity is antagonized by the anti-inflammatory cytokine IL-10. Our functional enrichment analysis also revealed alterations in IL-10 signaling with unloading, primarily due to an increase in the IL-10 receptor (IL-10RA) but not IL-10 itself. This is likely to be a compensatory mechanism in response to increased pro-inflammatory signaling, as is the increase in IL-1 receptor antagonist (IL-1RN) expression in the unloaded FAPs.

We compared our results with those of Oprescu and colleagues et al (Oprescu et al., 2020) who also performed RNAseq on FAPs. 15 genes overlapped in the “activated FAP” group between the two studies, 50% more than any other category (Table 2). These genes include inflammatory chemokines and cytokines (Cxcl5, Cxcl14, Cxcl12, IL33, and IL1r1); intracellular zinc storage (Mt2, Mt1); and genes involved with remodeling of the extracellular matrix (Mmp3; Timp1). The CXC family of chemokines is known to attract neutrophils and other immune cells to an inflamed area (Wuyts et al., 1998). IL-33 is a cytokine that, in muscle, promotes M2 macrophage differentiation (Hussey et al., 2019) and most likely coordinates with eosinophils through the IL-2 complex to promote induction of IL-5 which is needed for proper muscle regeneration (Kastenschmidt et al., 2021). IL1r1 is upregulated in sarcopenic muscle, and may be associated with lower muscle strength (Patel et al., 2014). Inhibition of the metallothioneins (Mt1 and Mt2) improves muscle strength and induces hypertrophy (Summermatter et al., 2017). High MMP3 and Timp1 levels have also been shown to be a potential biomarker for lean mass in elderly bed rest patients (Gawel et al., 2020). Upregulation of these genes, as seen in our results, contributes to an inflammatory microenvironment favorable to muscle atrophy (Table 3).

**TABLE 2:** Genes from RNA-seq results that overlap with different FAP cell categories. Genes that appeared in RNAseq data from each FAP category identified by Oprescu et al. (2020). The category of FAPs with the most overlapping genes was activated FAPs, with 15 genes, followed by Wisp1 FAPs with seven genes.

	Cxcl14 FAPs	Dpp4 FAPs	Dlk1 FAPs	Osr1 FAPs	Wisp1 FAPs	Activated FAPs	Fibroblasts
	Cxcl14	Igfpb5	Col3a1	Col3a1	Cthrc1	Cxcl5	Col3a1
	Apod	Gfpt2	Col1a1	Apod	Lox	Prg4	Col1a1
		Pla1a	Col1a2	Cxcl14	Sfrp1	Timp1	Col1a2
		Mt2	Igfbp7	Igfbp7	Timp1	Mt2	Mt2
		Efhd1		Col1a2	Angptl4	Mmp3	Igfbp7
		Dpp4			Cxcl12	IL33	
					Lrrc15	Cxcl14	
						Mt1	
						Lox	
						Npm1	
						Cxcl12	
						Gfpt2	
						Il1r1	
						Angptl4	
						Csgalnact1	
Total #	2	6	4	5	7	15	5

**TABLE 3:** List of activated FAP genes from RNA-seq data. Below are the fifteen activated FAP genes from Table 2 that overlapped with the RNA-seq data. All but one gene (Gfpt2) were upregulated significantly.

Gene Symbol	P-value (Left vs. Right)	FDR step up (Left vs. Right)	Fold change (Left vs. Right)	FDR step up (Leg * Gender)
Angptl4	7.96E-04	9.23E-02	5.05E+00	8.55E-01
Csgalnact1	2.80E-02	4.62E-01	2.51E+00	7.46E-01
Cxcl12	3.87E-05	8.98E-03	3.67E+00	9.06E-01
Cxcl14	1.36E-02	3.64E-01	3.59E+00	9.58E-01
Cxcl5	1.18E-02	3.39E-01	1.64E+02	7.39E-01
Gfpt2	5.26E-03	2.46E-01	-2.10E+00	9.89E-01
Il1r1	3.67E-04	5.37E-02	2.33E+00	9.08E-01
Il33	3.28E-02	4.84E-01	2.06E+00	8.95E-01
Lox	5.97E-04	7.64E-02	3.16E+00	9.03E-01
Mmp3	4.09E-03	2.21E-01	1.15E+01	8.33E-01
Mt1	1.38E-02	3.67E-01	2.58E+00	7.53E-01
Mt2	4.47E-02	5.26E-01	3.71E+00	8.19E-01
Npm1	5.01E-03	2.40E-01	2.02E+00	7.80E-01
Prg4	2.25E-02	4.35E-01	2.79E+00	7.46E-01
Timp1	3.39E-02	4.89E-01	6.54E+00	6.61E-01

Functional enrichment analysis of our RNA-seq data revealed significant upregulation of the IL-1β pathway in FAPs with immobilization (Figure 4). Whole muscle homogenates have been shown to have increased IL-1β after exercise (Dennis et al., 2004; Jensen et al., 2020), hindlimb immobilization (Caron et al., 2009) and traumatic injury *in vivo* (Kohno et al., 2012). Treatment of C2C12 myotubes with IL-1β impairs maturation and increases muscle cell catabolism (Li et al., 2009). IL-1β has also been implicated in muscle wasting disorders such as cancer cachexia (Graziano et al., 2005; Zhang et al., 2007; Deans et al., 2009; Suzuki et al., 2013; Fogelman et al., 2017), dysferlinopathy (Cohen et al., 2015; Hogarth et al., 2019) and Duchenne muscular dystrophy (Nagata et al., 2017). Importantly, Madaro and colleagues et al (Madaro et al., 2018) detected increased IL-1β in FAPs after denervation, further suggesting that IL-1β is a component of the FAP secretome that is stimulated with disuse. Most recently, Vumbaca and colleagues showed that IL-1β played an important role in the skeletal muscle secretome (Vumbaca et al., 2021). The FAP secretome includes some factors that are anabolic such as WISP1, which induces satellite cell expansion and differentiation (Lukjanenko et al., 2019; Zhang et al., 2020), and follistatin, which induces myoblast differentiation and inhibits myostatin (Amthor et al., 2004; Mozzetta et al., 2013). Our data underscore the fact that FAPs play a critical role in supporting muscle mass and mass function via their secretome, and that in certain settings such as disuse and immobilization the secretome can promote muscle atrophy through factors such as IL-1β.

In our immobilization model we also saw an absolute decrease in the number of FAPs compared to the non-immobilized limb, and a slight but non-significant decrease when FAP cell number is normalized to total cell count (Supplementary Material and Supplementary Table S2). This is surprising as FAPs normally increase in conditions such as ischemia-reperfusion or muscle injury (Joe et al., 2010; Heredia et al., 2013; Mozzetta et al., 2013; Madaro et al., 2018; Santini et al., 2020). This rapid proliferation allows FAPs to migrate to sites of injury and secrete factors that stimulate satellite cell proliferation and division. FAP cell number is, however, known to decline with aging (Josephson et al., 2019; Uezumi et al., 2021). Thus, the immobilization model may recapitulate some of the molecular and cellular changes that occur with aging and sarcopenia. Consistent with this interpretation we detected increased expression of the senescence marker Cdkn2a (p16) in FAPs from immobilized muscles. Recent studies have shown that FAPs can undergo senescence (Saito et al., 2020), and IL-1β is a well-established component of the senescence-associated phenotype (SASP) which partly explains it’s role in cancer cachexia (Lau et al., 2019). Results from our study therefore suggest that immobilization and disuse may contribute directly to muscle atrophy by inducing senescence and IL-1β expression in FAPs. Future research might be directed at targeting FAPs specifically using novel technologies such as capsid variants of adeno-associated viruses (AAVs; Tabebordbar et al., 2021) to alter gene expression in conditions of disuse atrophy to preserve muscle and function.

## Data Availability

The datasets presented in this study can be found in online repositories. The names of the repository/repositories and accession number(s) can be found below: https://www.ncbi.nlm.nih.gov/bioproject/ PRJNA787281.
