# Supplementary material for: Hindlimb Immobilization Increases IL-1β and Cdkn2a Expression in Skeletal Muscle Fibro-Adipogenic Progenitor Cells: A Link Between Senescence and Muscle Disuse Atrophy
Source: Front Cell Dev Biol. 2022 Jan 3;9:790437. doi: 10.3389/fcell.2021.790437 (PMC8762295; doi:10.3389/fcell.2021.790437)
Supplement: Supplementary file 2 [file DataSheet1.docx]

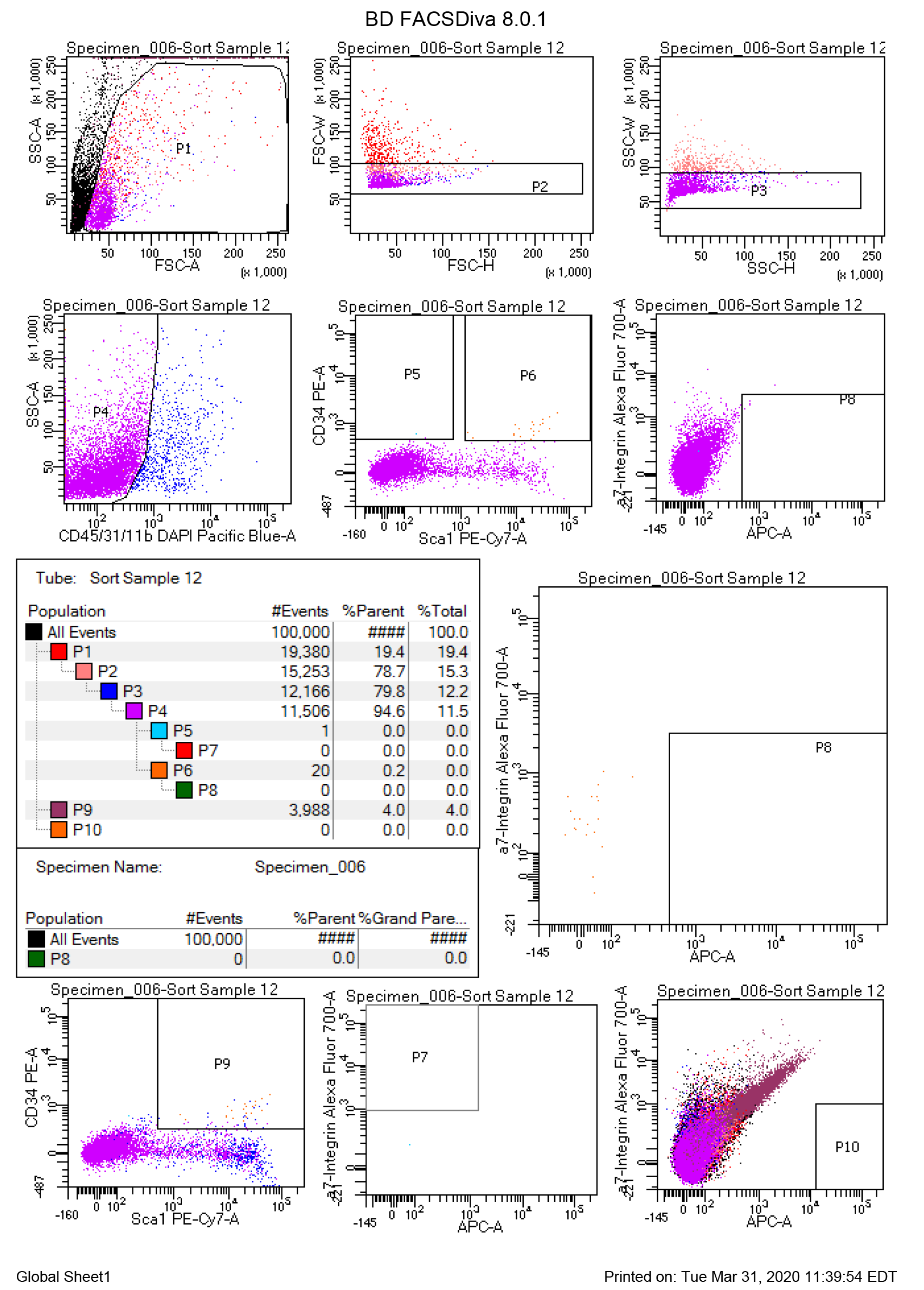


**Supplementary Figure 1. Gating used for FACs sorting.** Gating parameters used for the FACs sorts for FAP cells. P1 gates against cells positive for CD11b, CD31, CD45 and Dapi. The cells that are not positive for any of those markers then go to gate 6 which will select for cells positive for CD34 and Sca1. P8 gate is the gate of interest where cells positive for CD34, Sca1, CD140a and negative for α7-integrin (along with CD45,31,11b) are collected. On average 0.1-0.2% of the total cell population is collected.


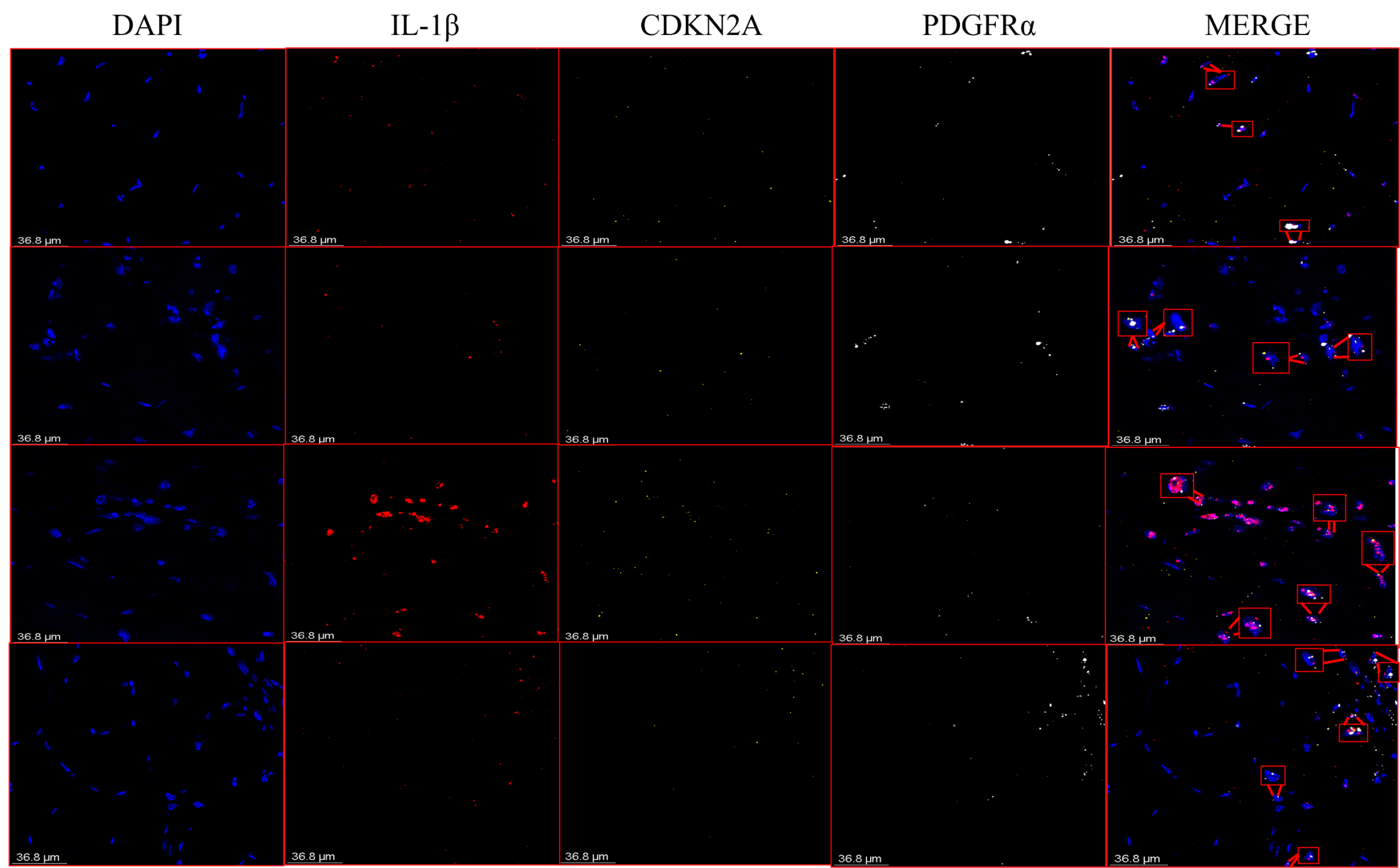


**Supplementary Figure 2. Channel images of RNAscope for immobilized muscle.** This figure shows the individual channels for staining of RNAscope of immobilized muscle for IL-1β (red), Cdkn2a (yellow) and PDGFRα (white) staining.


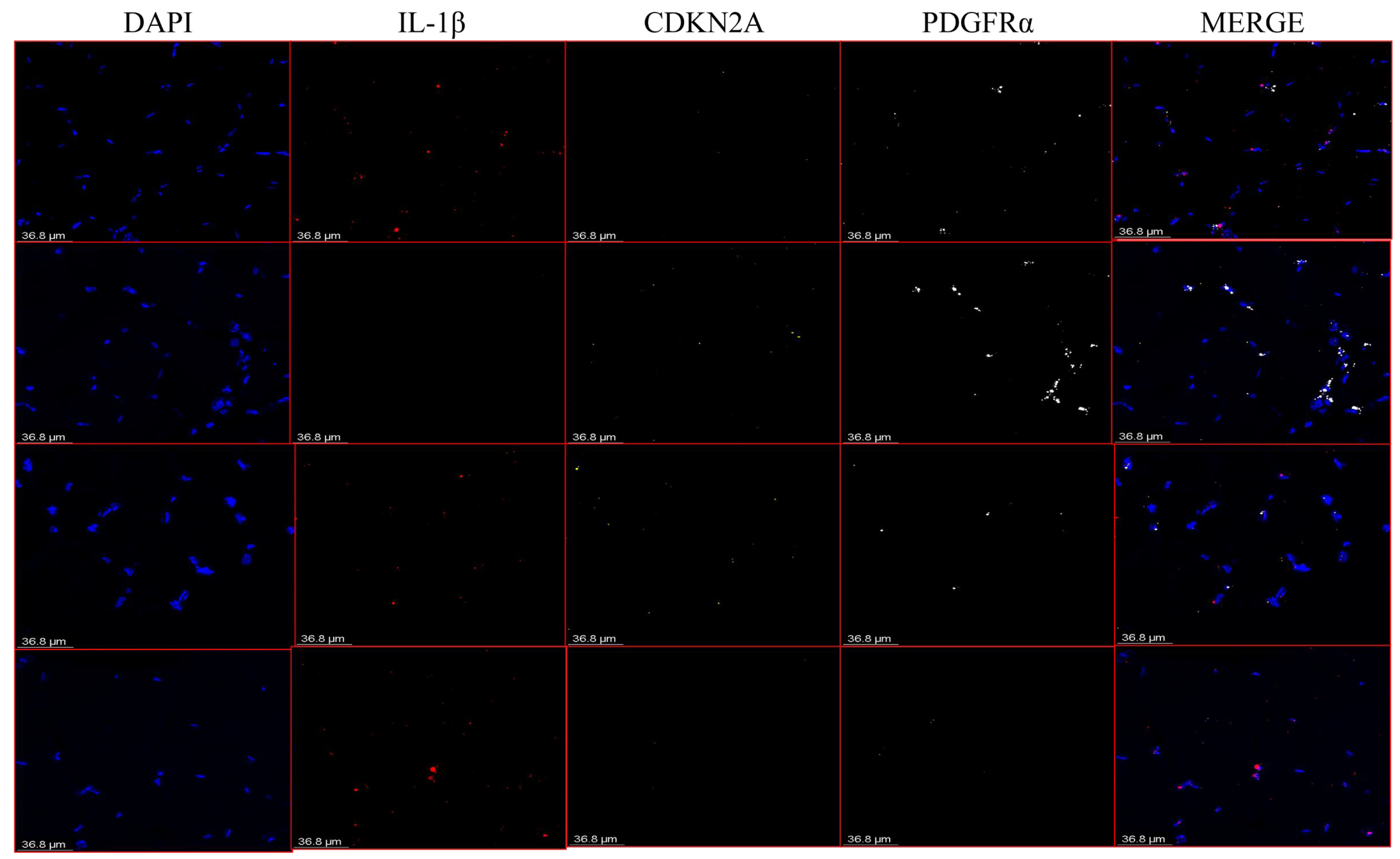


**Supplementary Figure 3. Channel images of RNAscope for nonimmobilized muscle.** This figure shows the individual channels for staining of RNAscope of the corresponding nonimmobilized muscle for IL-1β (red), Cdkn2a (yellow) and PDGFRα (white) staining to supplementary figure 2.


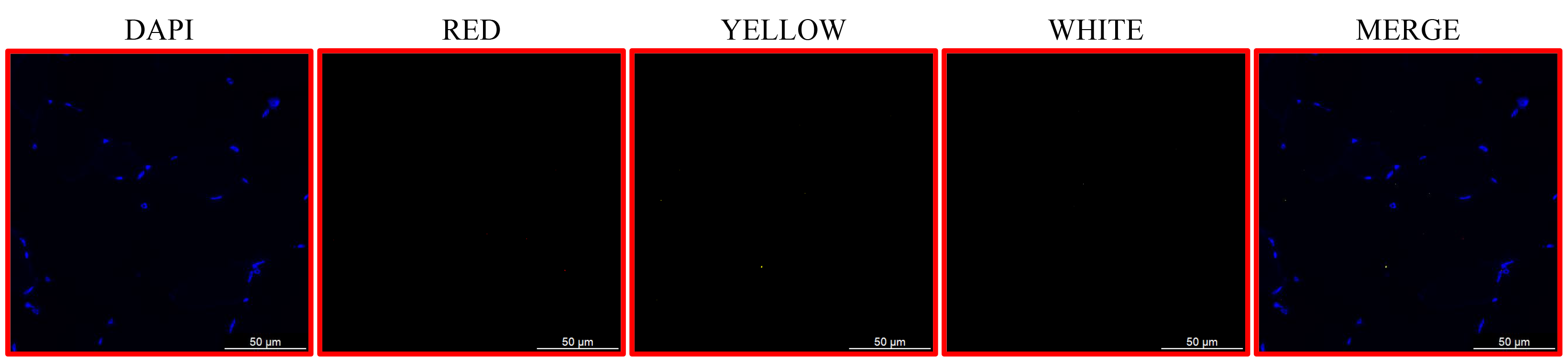


**Supplementary Figure 4. Negative control images for RNAscope staining.** This figure shows the negative control images taken for each channel. The first panel shows Dapi, followed by red, yellow and white color channels. The final panel shows the merged images. The negative control used for the staining process is for the DapB gene, a bacterial gene, from ACD bio.

**Supplementary Table 2. Number of FAP cells isolated from each sample.** Listed is the total number of FAP cells isolated from the sample via FACs as well as the total cells sorted through

| Sample | Immobilized | | Nonimmobilized | |
| --- | --- | --- | --- | --- |
|  | FAPs | Total Cells | FAPs | Total Cells |
| 1 | 1287 | 200000000 | 4155 | 330000000 |
| 3 | 2032 | 140000000 | 5698 | 430000000 |
| 4 | 6373 | 320000000 | 6666 | 570000000 |
| 5 | 5343 | 420000000 | 16000 | 3300000000 |
| 6 | 5058 | 380000000 | 15000 | 450000000 |
| 7 | 2614 | 32000000 | 2526 | 41000000 |
| 8 | 1611 | 23000000 | 3833 | 37000000 |
| 9 | 3032 | 30000000 | 1567 | 26000000 |
| 10 | 1332 | 14000000 | 6781 | 23000000 |
| 11 | 1982 | 30000000 | 4578 | 17000000 |
| 12 | 1740 | 13000000 | 4725 | 21000000 |
